# Comparative Studies on Leachability of Zinc and Iron from High-Energy Milled Waste of Scrap-Based EAF Steelmaking

**DOI:** 10.3390/molecules30204055

**Published:** 2025-10-11

**Authors:** Ewa Rudnik, Michał Stępień, Piotr Palimąka

**Affiliations:** Faculty of Non-Ferrous Metals, AGH University of Krakow, Al. Mickiewicza 30, 30-059 Krakow, Poland; mstepien@agh.edu.pl (M.S.); palimaka@agh.edu.pl (P.P.)

**Keywords:** ball milling, EAF waste, franklinite, leaching, mechanical activation, mechanochemical synthesis, recovery

## Abstract

Electric arc furnace (EAF) waste, a mixture of dust and slag, was investigated as a potential secondary source of zinc. The waste primarily consisted of zinc and iron oxides, with the presence of refractory zinc ferrite, which hinders the complete recovery of zinc. This is the first study that examined the effect of mechanical treatment through high-energy planetary ball milling on the phase transformation, metal speciation, and leachability of the EAF waste. The raw material was characterized by particle size distribution, morphology, phase composition, and sequential extraction, and then subjected to milling at different rotation rates (100–400 rpm). The resulting powders were analyzed using XRD, SEM–EDS, and sequential leaching, and tested for acid (H_2_SO_4_) and alkaline (NaOH) leachability. Milling progressively reduced particle size, increased surface roughness, and induced structural changes, including the mechanical activation effect at low milling rates (100 rpm) and the synthesis of secondary franklinite at higher milling energies (200 rpm and 400 rpm). Sequential extraction revealed changes in zinc and iron speciation from acid-soluble to residual fractions for increased milling intensities. Leaching experiments showed rapid zinc dissolution in both acidic and alkaline solutions, while iron dissolved only in acid. The highest zinc extractions (67% in H_2_SO_4_, 55% in NaOH) were obtained from mechanically activated material at 100 rpm, while zinc leachability decreased for higher milling rates due to the induced mechanical synthesis of refractory phase. The kinetic model of leaching of the main components of the EAF was also established.

## 1. Introduction

Zinc and iron are among the most important technical metals, alongside aluminum and copper [1,2]. Although these two metals are not officially classified as strategic or critical raw materials, zinc is recognized as a key resource in countries such as Canada [3], the United States [4], and Australia [5]. This significance is attributed to the broad range of zinc applications, which include traditional uses such as steel galvanizing and alloy production, as well as modern applications in advanced technologies involving precision die-casting, zinc–air batteries, and various components used in renewable energy systems [1]. As a result, zinc is forecast to become the fourth most valuable metal in the global market for selected critical materials between 2027 and 2030, preceded only by copper, lithium, and nickel [6]. Iron and steel, in turn, remain essential materials for infrastructure development and play a vital role in the transition toward a sustainable economy, serving mainly as fundamental construction materials for technologies such as geothermal, wind, solar, nuclear, and hydropower systems [7].

The production of secondary zinc and iron (steel) is inherently interconnected, as galvanized steel products such as car bodies or construction components are typically recycled in steel mills after reaching the end of their service life [8,9]. One of the commercially available technologies capable of utilizing up to 100% scrap for raw steel production is electric arc furnace (EAF) steelmaking [7]. This process operates by heating the metallic charge with an electric arc formed between graphite electrodes, enabling the molten material to reach temperatures of 1400–2000 °C [10]. Although EAF steelmaking accounted for only 29% of global crude steel production in 2023 [11], it is considered a key technology in the shift toward low-carbon metallurgy, as it emits more than three times less CO_2_ per tonne of crude steel compared to the conventional blast furnace–basic oxygen furnace route [12]. Despite its environmental and energy-related advantages, the EAF process generates considerable amounts of solid by-products, mostly 10–30 kg of dust and 60–270 kg of slag per tonne of liquid steel produced [13]. These waste streams contain not only significant amounts of recoverable iron (20–35%), but also high concentrations of zinc (9–40%) [14,15,16].

Electric arc furnace dust (EAFD) is the most important by-product of steel scrap processing, regardless of its classification as hazardous waste [13]. It is often further treated for zinc recovery using pyrometallurgical techniques, with the Waelz kiln being the most widely employed method [8,14]. Various hydrometallurgical or combined pyro-hydrometallurgical approaches have been also investigated, although their application at commercial scale remains limited [15,16]. The main drawback of hydrometallurgical treatment lies in the relatively low efficiency of acid or alkaline leaching (atmospheric or pressure), which typically achieves 50–80% zinc recovery [16]. This is due to the presence of zinc in the EAFD not only as easily leachable oxide ZnO (2–30%) but predominantly as refractory franklinite ZnFe_2_O_4_ (10–65%). To mitigate this issue, EAFD containing zinc ferrite has been proposed for conversion into more soluble phases prior to leaching [15,16]. Thus, the pretreatment methods included roasting with calcium oxide [17,18,19] or cement dust [20] to form leachable zinc oxide ZnO and insoluble calcium ferrite CaFe_2_O_4_; roasting with ammonium sulfates to produce soluble zinc sulfate ZnSO_4_ [21,22]; fusion with sodium hydroxide NaOH to yield soluble sodium zincate Na_2_ZnO_2_ [23,24]; and thermal treatment with polymers such as polyvinyl chloride PVC [25] or tetrabromobisphenol A TBBPA [26] to generate water soluble zinc compounds like zinc chloride ZnCl_2_ or potassium tetrabromozincate K_2_ZnBr_4_, respectively. In such cases, the leachability of zinc in various media like NaOH [17,18,20,23,24] or water [21,22,25,26] exceeded 80%.

Alternatively, pretreatment may involve mechanical or mechanochemical activation, where intensive high-energy grinding reduces grain size and can induce structural or chemical transformations in the material [27]. During this mechanical process, particles are deformed, repeatedly fractured and cold-welded through collisions with the milling balls and the chamber walls. Mechanical treatment of minerals [27] and secondary resources [28] enhances then leaching kinetics by increasing the surface area available to the leaching agent and, possibly, by activating surfaces, thereby enabling leaching at lower temperatures under atmospheric pressure. Moreover, when carried out in the presence of chemical additives, mechanochemical treatment can promote reactions within the material, leading to modifications in its chemical composition and, thus, its solubility. Although these methods have only occasionally been investigated with respect to industrial EAFD [29,30] or pure ZnFe_2_O_4_ [31], they undoubtedly demonstrate a beneficial effect on zinc leaching. For example, Zhang et al. [31] compared the alkaline leachability (6 M NaOH, 90 °C, 12 h) of mechanically activated ZnFe_2_O_4_ and mechanochemically reduced ZnFe_2_O_4_ using iron powder. Both treatments were performed in a planetary ball mill with stainless steel balls (1–5 mm in diameter; ball-to-ferrite mass ratio of 25:1). After 4 h of mechanical activation, about 13% of zinc was leached, representing only a slight improvement compared with the untreated sample (near 2%). In contrast, mechanochemical reduction of ferrite increased zinc extraction to about 60% when the Fe/ZnFe_2_O_4_ ratio exceeded 2, and up to 70% after extended milling (6–12 h). This enhancement in zinc leachability was attributed to the decomposition of zinc ferrite and the formation of Zn_x_Fe_3–x_O_4_ solid solutions, as XPS analysis revealed an increased Fe^2+^ content resulting from the reduction of Fe^3+^ by metallic iron. In other studies, the authors [30] investigated chemical and mechanochemical leaching of EAF dust (22% Zn, 23% Fe, 3.5% Pb). Mechanochemical leaching was performed directly in a stirred ball mill (5 M NaOH, 90 °C, 4 h, ball-to-EAFD mass ratio 30:1) and resulted in increased zinc leachability (45%) compared with conventional leaching of the non-activated sample (25%). In this case, no structural changes in the material were reported. In turn, Zoz et al. [29] proposed a two-stage process, consisting of mechanical activation followed by ammoniacal leaching, performed in the same high-energy ball mill but with horizontal and vertical orientations, respectively. During milling (20 min, 3 mm steel balls, ball-to-EAFD mass ratio 10:1, 1300 rpm), the particle size of EAFD (33% Zn, 27% Fe, 1.5% Pb) was reduced from 5 µm to 1 µm, generating numerous structural defects and increasing the reactivity of the dust. In the subsequent leaching step in ammonia–ammonium carbonate solution, it was observed that high-energy milling did not overcome the inherent resistance of zinc ferrite to the leaching agent; however, ZnO was fully dissolved within 0.5 h, compared with over 1 h for the untreated dust. As in previous studies, no structural changes in the milled material were reported.

As discussed above, the literature data on mechanical activation of EAF waste and its effects on structural changes and zinc leachability remains very limited. To address this gap, the present study was undertaken, focusing on phase transformations in mechanically activated EAF waste, changes in metal speciation in the material, and metal leaching under both acidic and alkaline conditions. The aim of this work was to evaluate the effect of ball milling at different rotation speeds on structural changes in the material, and to establish correlations between the milling parameter and leaching behavior of key metals.

## 2. Results

### 2.1. Raw Material

**Figure 1:**
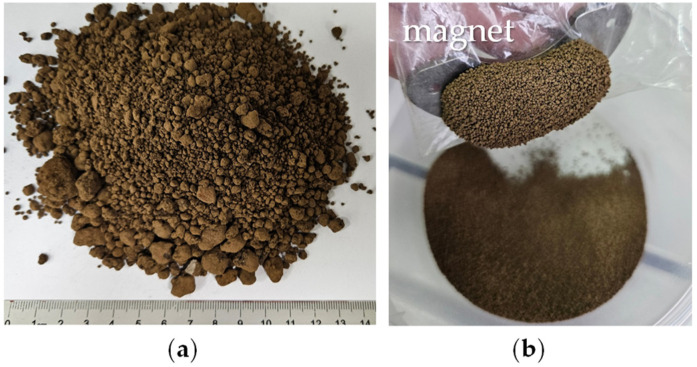
(**a**) Macroscopic view of the raw material; (**b**) Magnetic behavior of the powder.

**Figure 2:**
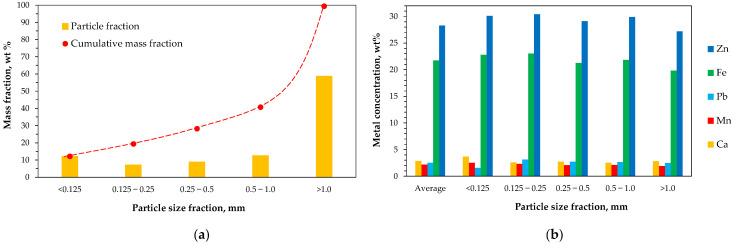
(**a**) Particle size distribution of the EAF waste; (**b**) Concentrations of the main metals in the raw material and its individual particle-size fractions. Values represent mean data (*n* = 3).

The raw material was collected from a Polish landfill of electric arc furnace residues. It was a red-brown powder with a heterogeneous particle size distribution and magnetic properties (Figure 1). Sieve analysis of the material into five particle-size fractions revealed that coarse particles larger than 1 mm represented the dominant fraction, accounting for about 60%, while the remaining finer fractions contributed 5–10% each (Figure 2a).

Wet chemical analysis of each fraction revealed relatively uniform percentages of the main metals. Zinc and iron were the predominant components, with average contents of 28.3% and 21.5%, respectively. The other analyzed metals, such as lead, manganese, and calcium, accounted for about 2–3% each (Figure 2b).

**Figure 3:**
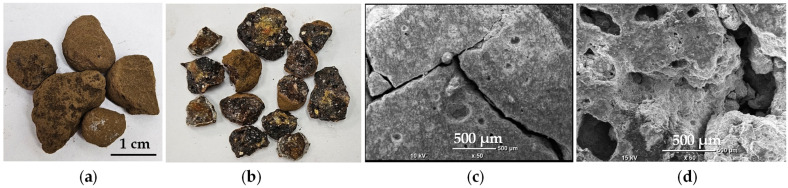
Macroscopic view of the large particles (**a**) and their cross-sections (**b**); (**c**,**d**) SEM micrographs of particle cross-sections.

**Figure 4:**
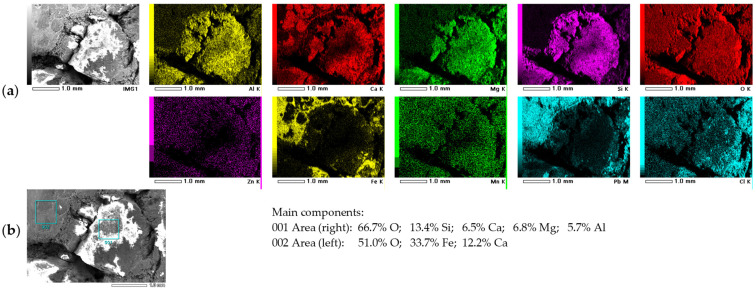
(**a**) Elemental distribution on the cross-section of large EAF raw material particles; (**b**) Local chemical composition.

A detailed morphological analysis of the largest particles, with diameters on the order of 0.5–1.5 cm (Figure 3), revealed a cracked and porous material, typical of EAF slag residues. The slag formed on the surface of the molten bath in the electric arc furnace solidifies upon cooling into a porous, rock-like material, with the porosity resulting from carbon oxides generated by the reaction of carbon in the steel scrap with oxygen at high temperatures [16]. EDS analysis of the particle cross-section confirmed the presence of elements characteristic for the slag, such as aluminum, calcium, magnesium, silicon, and oxygen. Elemental mapping further revealed uneven distribution of these elements (Figure 4a), while local analysis indicated the presence of calcium and magnesium aluminosilicates and calcium ferrite CaFe_2_O_4_ (Figure 4b). Noteworthy, typical crystalline components of the EAF slag are larnite Ca_2_SiO_4_, wollastonite CaSiO_3_, gehlenite Ca_2_Al(AlSi)O_7_, bredigite Ca_7_Mg(SiO_4_)_4_, brownmillerite Ca_2_(Al,Fe)_2_O_5_, diopside CaMg(SiO_3_)_2_ [32]. Nevertheless, none of these calcium compounds was further confirmed in detectable amounts in the investigated raw material, except CaFe_2_O_4_.

**Figure 5:**
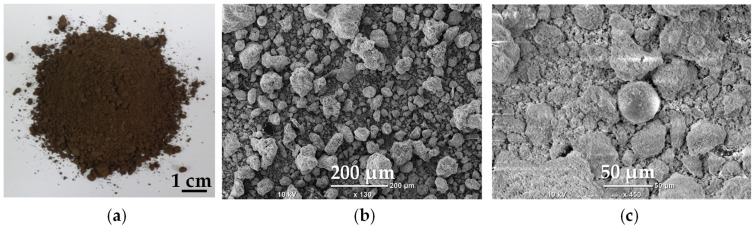
(**a**) Macroscopic view of the finest particle fraction; (**b**,**c**) SEM micrographs of the particles.

The finest particle fraction (below 0.125 mm) of the raw material consisted of particles of various shapes (irregular, sphere-like) and sizes, featuring a characteristic rough surface (Figure 5). EDS analysis revealed that the main components of this fraction are zinc (35%), iron (25%), oxygen (25%), and manganese (3%), which are typical for EAF dust [16]. Other elements usually identified in the EAF dust, such as silicon (1.5%), aluminum (0.5%), and magnesium (0.4%), were present only in trace amounts.

It is worth noting that the waste material was not a simple mixture of fine particles of pure EAF dust (collected from the de-dusting system) and larger particles of pure slag (taken from the furnace). The EAF waste originated from a storage site where these two materials had been mixed, allowing for crushing and/or the formation of secondary granules of dust and/or slag. As a result, the elemental contents in the individual particle-size fractions were essentially comparable.

**Figure 6:**
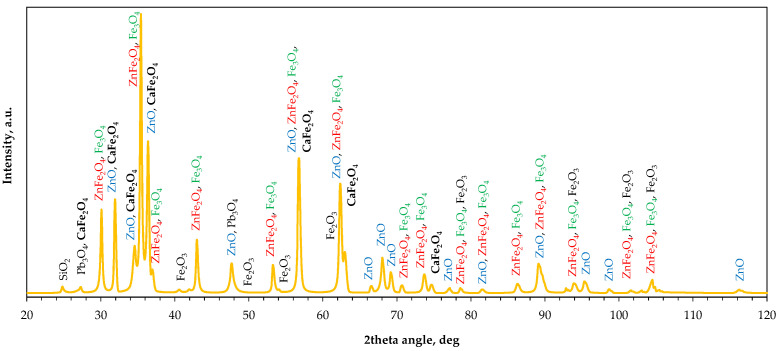
X-ray diffraction pattern of the raw material.

X-ray diffraction analysis of the raw material (Figure 6) revealed a mixture of multiple phases, typical for EAF dust contaminated with slag particles [33,34]. The main identified compounds include zincite ZnO, franklinite ZnFe_2_O_4_, and magnetite Fe_3_O_4_, with possible traces of calcium ferrite CaFe_2_O_4_, hematite Fe_2_O_3_, lead oxide Pb_3_O_4_ and silica SiO_2_. It is important to note that the phase composition of the EAF waste is somewhat complex, consisting of different crystalline components whose overlapping diffraction peaks make unambiguous phase identification faintly problematic.

### 2.2. High-Energy Milled Materials

The raw material was subjected to grinding in a high-energy planetary mill equipped with tungsten carbide balls. The milling was carried out in the presence of water at three different rotation speeds, ranging from 100 rpm to 400 rpm. The obtained samples were then compared with the original material in terms of morphology and composition to identify changes that could influence subsequent leaching behavior.

#### 2.2.1. Morphology

**Figure 7:**
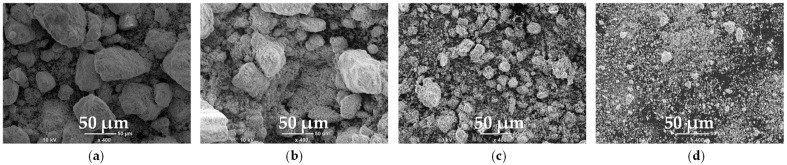
SEM micrographs of the raw material (**a**) and samples after mechanical treatment at rotation rates of: (**b**) 100 rpm, (**c**) 200 rpm, (**d**) 400 rpm.

Figure 7 presents the morphology of the raw material and the samples subjected to mechanical treatment. With increasing rotation rates, and thus higher milling energy, a gradual reduction in particle size was observed, with the most pronounced effects obtained at 200 rpm and 400 rpm. At the latter condition, the material was transformed almost entirely into a powdery form. Milling at 100 rpm had a minor effect on particle size; however, a distinct increase in surface roughness was noticed. This enhanced roughness may be attributed to the formation of finer particles on the surfaces of larger ones. It is noteworthy that the mineral components of EAF waste can be ground to varying degrees depending on their hardness, which increases in the following order: ZnO (150–180 HV [35]) < ZnFe_2_O_4_ (200–470 HV [36]) < Fe_3_O_4_ (500–600 HV [37]) < CaFe_2_O_4_ (800–900 HV [37]) < Fe_2_O_3_ (1000 HV [37]).

#### 2.2.2. Elemental Composition

**Figure 8:**
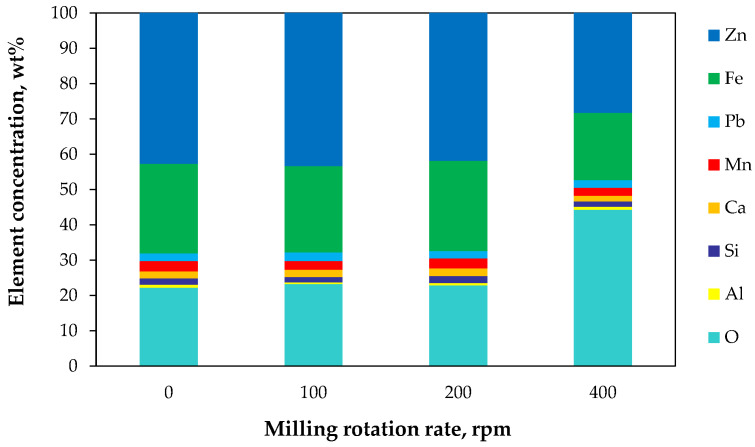
Effect of high-energy milling rotation rates on elemental composition of EAF waste (EDS surface analysis).

The powders before and after mechanical treatment were investigated by EDS to compare the elemental composition of the materials. The analyses were performed in different surface areas, and the results are presented in Figure 8. For the raw material sample (0 rpm), zinc and iron concentrations were found to be about 15 wt% and 5 wt% higher, respectively, than those obtained by wet chemical analysis (Figure 2b). In contrast, the EDS results for the raw sample were consistent with those obtained for the fine raw material fraction. The difference between the results of spectroscopic and wet analyses is understandable since EDS provides information only from the surface layer of EAF particles (locally), whereas chemical dissolution reflects the average bulk composition. It should also be emphasized that EAF dust and slag particles exhibit distinct surface and bulk compositions, which result from the reaction mechanisms during the formation of both wastes. For example, Suetens [38] demonstrated a zinc concentration gradient within iron oxide particles in EAF dust, with zinc content decreasing from the particle surface toward its core. On the other hand, compositional heterogeneity is visible directly in slag grains, whose surfaces are covered with iron oxide residues (Figure 3b), while their centers display an uneven distribution of elements (Figure 4).

Regardless of the above considerations, a detailed analysis of the elemental composition of the powders shows that the composition of the materials changes gradually with increasing milling rate. This effect is most pronounced at the highest rotation speed (400 rpm), where the oxygen content doubles compared to the other samples. At the same time, the proportions of zinc and iron shift toward values characteristic of the average bulk composition (Figure 2b). The gradual abrasion of surface layers, followed by intensive particle fragmentation during milling, confirms the heterogeneity of the particle components and induced elemental changes. However, partial secondary oxidation of the intensely ground powder cannot be ruled out as the milling process generates elevated temperatures.

#### 2.2.3. Phase Composition

**Figure 9:**
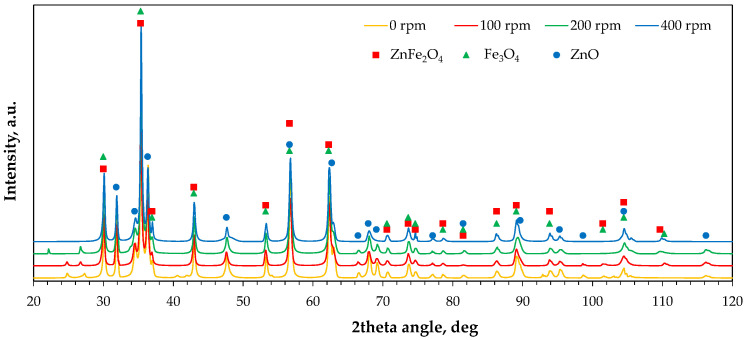
X-ray diffraction patterns of the raw (0 rpm) and high-energy milled (100–400 rpm) EAF waste.

**Figure 10:**
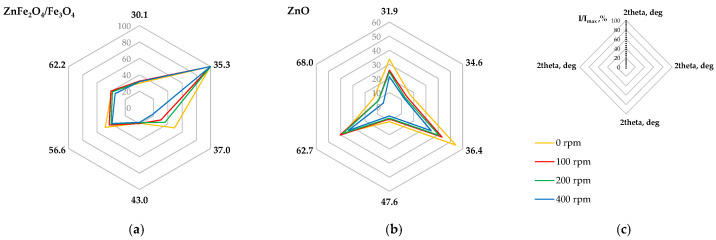
Changes in relative intensities of diffraction peaks of main phases of the raw (0 rpm) and high-energy milled (100–400 rpm) EAF waste: (**a**) ZnFe_2_O_4_/Fe_3_O_4_, (**b**) ZnO, (**c**) plot legend.

Figure 9 shows the X-ray diffraction patterns of the raw and milled powders. The main zinc- and iron-containing phases were identified, but changes in the relative intensity of their characteristic peaks were observed. Figure 10 shows radar plots illustrating changes in the relative intensities of six peaks of the main phases, namely ZnFe_2_O_4_ with Fe_3_O_4_ (due to possible peak overlapping at characteristic diffraction angles) and ZnO. The relative peak intensity was calculated as I/I_max_, where I_max_ is the intensity of the highest diffraction peak, assumed as 100% (i.e., the peak at a 2θ angle of 35.4°, collective for ZnFe_2_O_4_/Fe_3_O_4_). It was found that increasing milling rate gradually decreases the peak intensities of ZnO, with the largest shift observed between 0 rpm and 100 rpm (Figure 2b). In the case of franklinite/magnetite, only three peaks disappear significantly: at 2θ of 37° and, to a lesser extent, at 2θ values of 56.6° and 62.2°. Interestingly, the peak at 37° disappears in the following order of milling rates: 0 rpm > 200 rpm > 100 rpm > 400 rpm, whereas for the 62.2° angle the changes follow the order: 0 rpm = 100 rpm > 200 rpm > 400 rpm (Figure 2a). As the peak intensities are proportional to the phase contents in the solid material, these variations indicate possible transformations among the components, such as the formation of a hardly soluble Fe_3_O_4_–ZnFe_2_O_4_ solid solution [39] or the reaction of ZnO with Fe_2_O_3_ leading to ZnFe_2_O_4_ synthesis [40] at the highest milling energy, and the accompanying structural deformation of franklinite [41].

#### 2.2.4. Sequential Leaching

**Figure 11:**
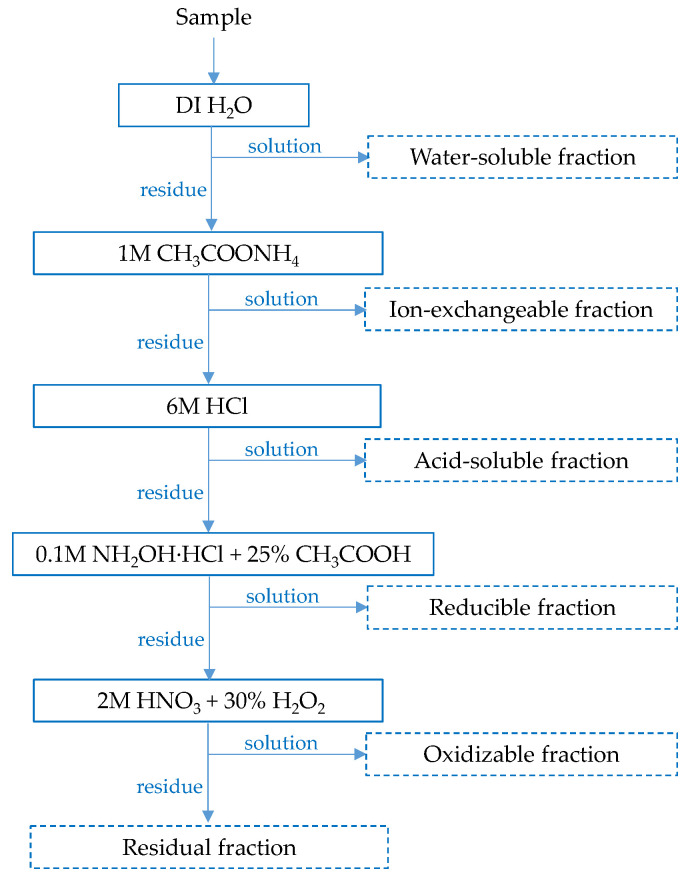
Scheme of sequential extraction of metals from the raw and high-energy milled EAF waste.

Sequential leaching procedures are most commonly employed to quantitatively determine the modes of occurrence of metals in minerals [42,43], although they have also been applied to zinc speciation in steelmaking dusts [44,45]. Sequential extraction involves the selective dissolution of metals in consecutive stages, identifying their existence as water-soluble, ion-exchangeable, reducible (e.g., iron–manganese oxides), acid-soluble (e.g., carbonates), oxidizable (e.g., organic matter and/or sulfides), and residual (e.g., silicates) forms. In this study, a combination of two procedures [42,46] was used, following the scheme shown in Figure 11. This method employed hydrochloric acid to selectively dissolve zinc and iron oxides while preserving franklinite, which remains stable at relatively low temperatures [47].

**Figure 12:**
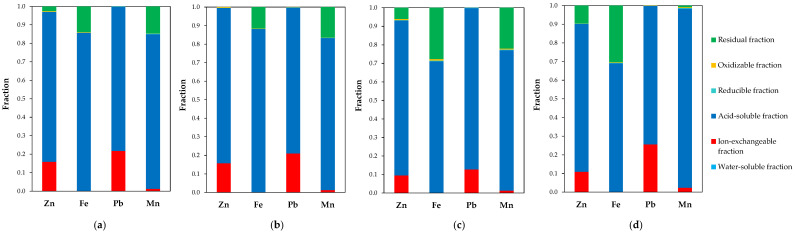
Modes of metal occurrence in (**a**) the raw material and high-energy milled at different rotation rates: (**b**) 100 rpm, (**c**) 200 rpm, (**d**) 400 rpm. Values represent mean data (*n* = 3).

Figure 12 presents the distribution of the main metals (Zn, Fe, Pb, Mn) among different fractions of the raw and milled material. It was found that none of the metals were present in the water-soluble fraction, while only traces were detected in the oxidizable and reducible forms. Some zinc and lead were extracted with ammonium acetate, which targets ion-exchangeable species; however, it is possible that part of the metal oxides reacted with the reagent due to the ready solubility of zinc and lead acetates. The majority of all metals were present in the acid-soluble fraction, especially since a relatively concentrated hydrochloric acid solution was used at this stage. Consequently, soluble chloride species (salts and/or complexes) were formed. The remaining amounts of metals, such as Zn, Fe, and Mn, were bound in the solid residue. For comparison, Lanzerstorfer and Preitschopf [45] reported zinc speciation in EAF dusts using a different sequential leaching procedure. They found that two-thirds of the total zinc was present as oxide (25%) and ferrite (i.e., 40% in the residual fraction), while the remaining third was attributed to carbonated (27%) and reduced (8%) fractions.

The effect of milling intensity on the speciation of zinc and iron is noticeable. As the rotation speed increases, their proportion in the residual fraction also increases, except for at 100 rpm. This may confirm the occurrence of mechanical synthesis and the transformation of some zinc and iron oxides into poorly leachable secondary franklinite.

**Figure 13:**
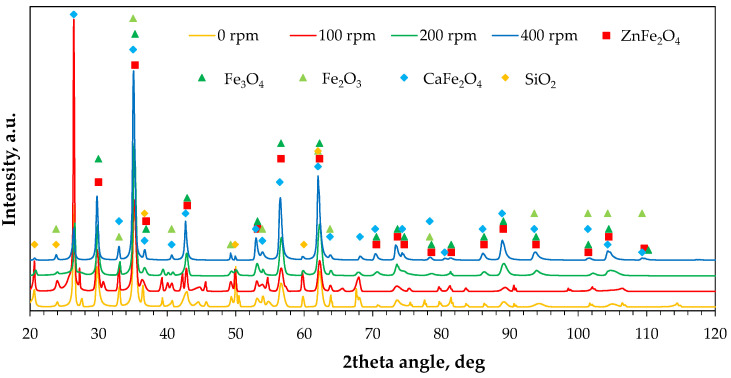
X-ray diffraction patterns of solid residues after sequential leaching of the raw (0 rpm) and high-energy milled (100–400 rpm) EAF waste.

Figure 13 shows X-ray diffractograms of the solid residues from the sequential extraction. The plot includes potential phases that could be present in the solids, though some peaks may overlap. These phases include franklinite, magnetite, hematite, calcium ferrite, and silica. No peaks associated with zinc oxide were detected. The behavior of iron oxides is challenging to interpret due to covering with peaks of other phases, but it seems that these iron phases are of minor importance. Nevertheless, significant changes are evident at the diffraction angle of about 30.3°. In the raw material, a distinct double peak is observed at 2θ = 35.14° and 2θ = 35.42°, corresponding to the main reflections of franklinite and magnetite, respectively. Upon milling, the magnetite peak disappeared, indicating its reaction with acid, while the franklinite peak remained at the same position, confirming its chemical resistance.

**Figure 14:**
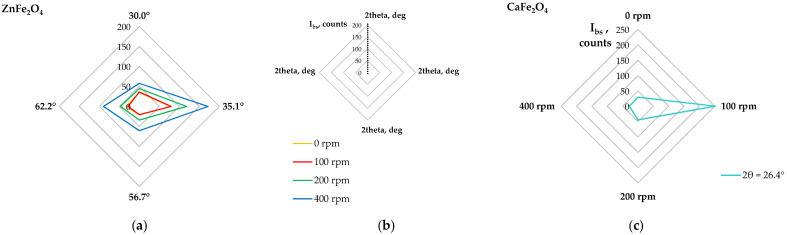
Changes in background-subtracted intensities I_bs_ of diffraction peaks of main phases of the solid residues after sequential leaching of the raw (0 rpm) and high-energy milled (100–400 rpm) EAF waste: (**a**) ZnFe_2_O_4_ at various 2θ, (**b**) plot legend, (**c**) CaFe_2_O_4_ at 2θ of 26.4°.

A closer examination of the identified peaks revealed that their intensities changed with increasing milling rates. The diffractograms were processed to obtain background-subtracted intensities, where the signal at non-diffracting angles equals zero. The radar plots shown in Figure 14a,b indicate that the peak intensities attributed to franklinite were the same for the raw material and powder milled at 100 rpm, but at higher milling rates, the intensity values significantly increased, especially at 400 rpm.

Interestingly, for the ball milling at 100 rpm, diffraction peaks corresponding to CaFe_2_O_4_ were the strongest, while at higher milling energies, these peaks diminished (Figure 13 and Figure 14c). These observations confirm that at the milling rate of 100 rpm, the EAF waste undergoes mechanical activation, facilitating the dissolution of zinc and iron oxides, while leaving ZnFe_2_O_4_ and CaFe_2_O_4_ largely unaffected. In contrast, higher milling energies promote the mechanical synthesis of secondary ZnFe_2_O_4_.

### 2.3. Acid and Alkaline Leaching

#### 2.3.1. Leaching

**Figure 15:**
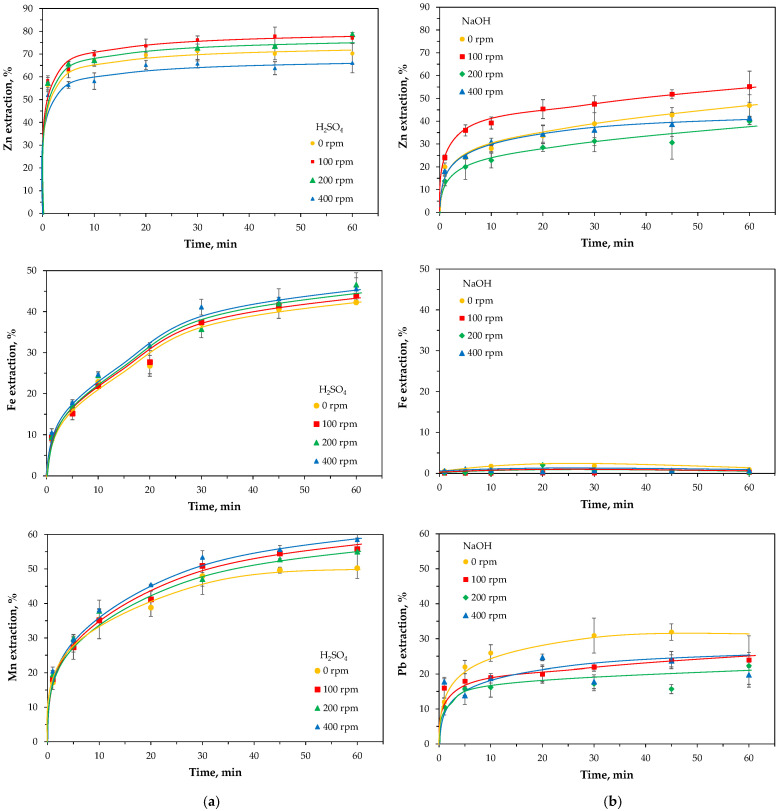
Effect of leaching time on metal extraction from the raw (0 rpm) and high-energy milled (100–400 rpm) EAF waste in: (**a**) 2 M H_2_SO_4_, (**b**) 4 M NaOH. Values represent mean ± standard deviation (*n* = 3).

The raw and milled powders were subjected to leaching experiments in sulfuric acid and sodium hydroxide solutions, each at the same concentration of active species (4 M H^+^ or OH^−^), to evaluate the effect of high-energy milling on metal solubility. Both reagents are considered classical leaching agents with lower aggressiveness compared to HCl used previously in the sequential extraction. H_2_SO_4_ is inexpensive and readily reacts with metal oxides, though it lacks selectivity toward zinc and iron oxides. In turn, NaOH selectively dissolves zinc oxide, without affecting the iron compounds. The experiments were carried out at a slightly elevated temperature (60 °C) and with a high liquid-to-solid ratio (L/S = 50) to minimize reagent consumption effects and ensure unobstructed reaction progress. Comparative results for both leaching conditions are presented in Figure 15.

As expected, clear differences were observed between the behavior of acidic and alkaline solutions, with the analyzed metals (Zn, Fe, Mn, Pb) exhibiting distinct leaching kinetics. In sulfuric acid, considerably higher extraction degrees were obtained compared to sodium hydroxide. Zinc dissolved very rapidly in both media, reaching its maximum extraction levels (65–78% in H_2_SO_4_, 40–55% in NaOH) within about 30 min, with a noticeable effect of milling rate. In contrast, iron leaching occurred only in acidic solution (42–47% in H_2_SO_4_, 0.1–0.9% in NaOH), and the milling rate had virtually no influence on its dissolution. Interestingly, the kinetic curves suggest a two-step dissolution process, with a distinct inflection after approximately 20–30 min. The leaching reagents showed selective action toward manganese and lead: manganese was leached gradually only under acidic conditions (50–58% in H_2_SO_4_, 0% in NaOH), whereas partial dissolution of lead oxide occurred principally in alkaline solution (1–3% in H_2_SO_4_, 20–24% in NaOH) reaching relatively stable concentrations levels within 10 min.

#### 2.3.2. Solid Residues

**Figure 16:**
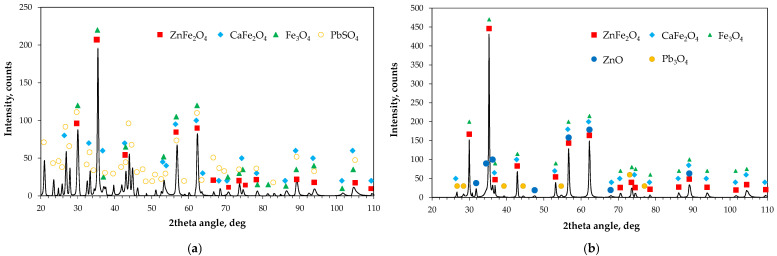
Typical X-ray diffraction patterns of solid residues after leaching of the raw and high-energy milled (100–400 rpm) EAF waste in: (**a**) 2 M H_2_SO_4_, (**b**) 4 M NaOH.

The solid residues collected after leaching were washed and dried. Phase analysis (Figure 16) showed that zinc and calcium ferrites, as well as magnetite, always remained either unreacted or only partially dissolved, regardless of the milling intensity or the type of leaching solution used. Zinc oxide readily reacted with acid, whereas in alkaline solution undissolved residues or secondary precipitates were detected. Lead oxide initially present in the material reacted with acid and then precipitated as lead sulfate PbSO_4_. In contrast, Pb_3_O_4_ appeared to be only weakly affected by NaOH, as it was identified in the leaching residues, which may explain the variability in lead ion concentrations observed in the kinetic curves.

**Figure 17:**
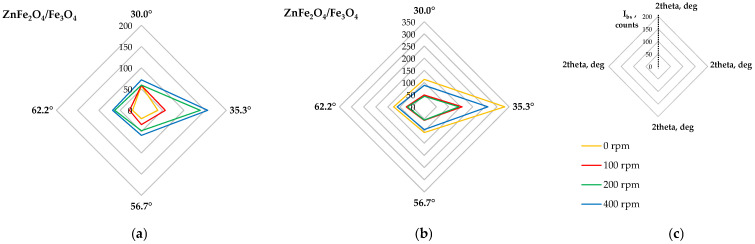
Changes in background-subtracted intensities I_bs_ of ZnFe_2_O_4_/Fe_3_O_4_ diffraction peaks of main phases of the solid residues of the raw (0 rpm) and high-energy milled (100–400 rpm) EAF waste after leaching in: (**a**) H_2_SO_4_, (**b**), NaOH, (**c**) plot legend.

Analysis of the background-subtracted diffractograms indicates that peak intensities depend on the milling rate. The radar plots (Figure 17) for four main 2θ angles attributed to franklinite/magnetite confirm structural changes occurring in the EAF waste during ball milling. Based on the different responses of ferrites and oxides to acidic and alkaline leachants, these changes can be inferred primarily from the diffraction peaks at about 30° and 35°, as well as from two additional peaks despite partial overlap of the latter with other phases. Sulfuric acid, being a non-selective reagent, reacts first with iron oxides Fe_2_O_3_ and Fe_3_O_4_ and may also slightly attack ZnFe_2_O_4_, as suggested by the bend on the kinetic curves. However, the intensities of the characteristic peaks of franklinite increased markedly (Figure 17a), confirming its growing fraction in the solid residues at 200 rpm and 400 rpm, i.e., at milling rates at which mechanosynthesis of secondary franklinite can take place.

In contrast, NaOH shows little reactivity toward franklinite and iron oxides, leaving them in the solid residue (Figure 17b). Although the effect of milling rate differs in this case, an evident increase in the intensity of franklinite peaks, and thus its fraction in the residue, is observed with higher milling intensities.

## 3. Discussion

The results of the experiments demonstrate the influence of mechanical treatment on the structural properties, speciation of EAF waste, and zinc leachability in both acid and alkaline solutions. However, due to the limited number of literature reports, it is difficult to compare the obtained data with other experiments. As mentioned in the Introduction, only one study [31] examined the effect of mechanical activation on zinc leaching from the EAF dust, but it involved extended mechanical treatment (4 h, no milling rate was shown) and more aggressive leaching conditions (6 M NaOH, 90 °C, 12 h). Moreover, no data are available regarding acid leaching of EAF dust after mechanical treatment.

**Figure 18:**
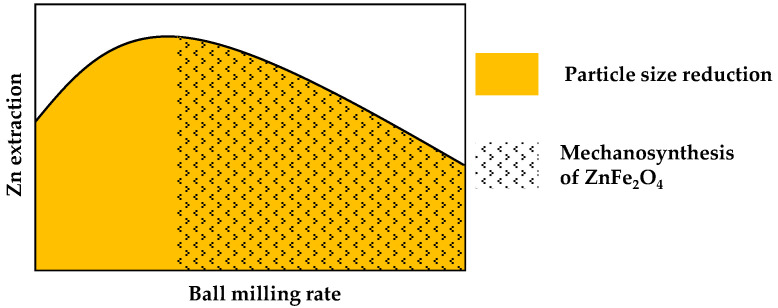
General scheme of changes in zinc extraction caused by structural transformations in EAF waste induced by increasing milling rate.

Nonetheless, the leaching effects were strongly dependent on milling intensity (Figure 18). At a low milling rate (100 rpm), mechanical activation was identified as the key factor behind the significant improvement in zinc leachability. At a medium milling rate (200 rpm), despite the crushing the solid particles, which positively influenced leaching results, an accompanying process began to play a role, leading to the formation of secondary refractory zinc ferrite. This, in turn, reduced the effectiveness of metal leaching. At the highest milling energy (400 rpm), intense secondary synthesis of franklinite occurred, and despite the significant reduction in material particle size, the resulting zinc extraction was lower compared to untreated EAF waste.

**Figure 19:**
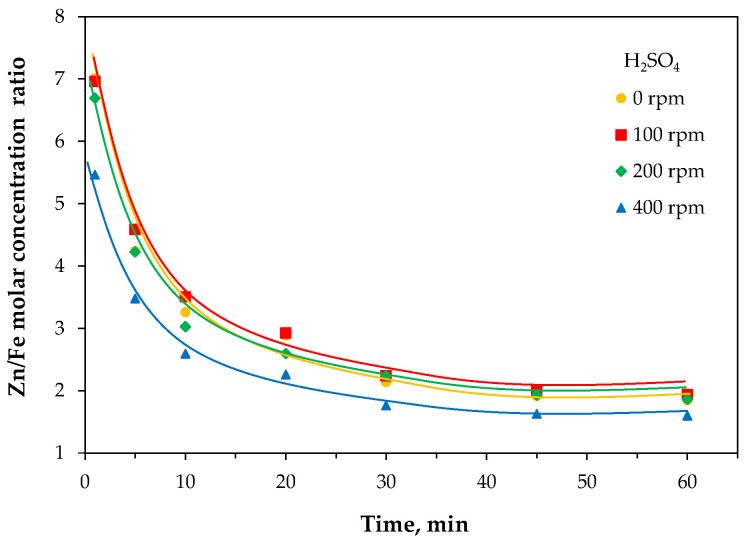
Time-dependent Zn/Fe molar concentration ratio during acid leaching of the raw (0 rpm) and high-energy milled (100–400 rpm) EAF waste.

Comparison of the molar concentrations of zinc and iron ions in the acid leaching solution (Figure 19) showed a preferential transfer of zinc from the EAF waste, which is understandable since iron oxides are more resistant to H_2_SO_4_ attack. The Zn/Fe concentration ratio was lower for the material milled at the highest rotation rate, confirming that the extraction of zinc was hindered by the changes induced in the EAF waste. Interestingly, in all cases, this ion concentration ratio decreased with time, reaching after 30 min stable values of 1.9 and 1.6 for milling rates of 0–200 rpm and 400 rpm, respectively. These ratios are somewhat higher than the Zn/Fe molar ratio of 1.1 in the solid EAF waste.

It is worth noting that the observed trends in the leaching kinetics of zinc and iron from EAF waste in H_2_SO_4_ are similar to the dissolution behavior of franklinite in the acid of comparable concentration. Tkáčová et al. [40] showed that the dissolution behavior of zinc ferrite prepared by mechanical synthesis was dependent on the acid concentration and in a 3 M solution, the Zn/Fe concentration ratio decreased over time, while in a 1 M solution, it increased, finally reaching a plateau at a Zn/Fe ratio of 1.05. Additionally, they reported that during the dissolution of non-activated ferrite, zinc dissolved first, followed by iron (Zn/Fe > 1). In contrast, when mechanically activated ferrite was dissolved, iron dissolved prior to zinc. This change was attributed to the transition of zinc ferrite into a metastable state due to mechanically induced (reversible) inversion and partial deformation of the anion sub-lattice in octahedral geometry. As the degree of inversion of the mechanically activated zinc ferrite increased, the rate of metal dissolution in acid also increased. Further studies [48] indicated that under mechanical activation, the normal spinel structure of zinc ferrite converts into a metastable inverse spinel structure, with the degree of inversion increasing with ball milling time. It was concluded that the mechanically induced reactivity of zinc ferrite results from its high degree of dispersity and structural metastability, which are associated with the mechanically induced disorder and the nanoscale nature of its structure. However, no further investigation of the leaching behavior was conducted.

On the other hand, Lumongsod et al. [39] revealed that ZnFe_2_O_4_ and Fe_3_O_4_ can form solid solutions in the EAF dust, with each component exhibiting distinct leachability in acids. To investigate this, they synthesized by high-temperature solid-state reaction a series of (1 − x)ZnFe_2_O_4_-xFe_3_O_4_ spinel solid solutions, where x denotes the molar percentage of magnetite. It was found that almost complete zinc dissolution in HCl (1 M, 2 h) was achieved only for x = 30% at an elevated temperature of 85 °C (while only 20% extraction occurred at 50 °C). The enhanced dissolution of the spinel solid solution at this composition was attributed to a transition from normal to inverse crystal structure for x in a range of 20–30% Fe_3_O_4_. The larger Fe^2+^ ions in both the tetrahedral and octahedral sites at this spinel inversion composition destabilized the crystal structure, weakening the Fe^2+^–O bonds, making them more susceptible to acid attack during leaching. Therefore, by the authors concluded that by modifying the EAF dust with Fe_3_O_4_ to achieve optimal composition of the solid solution, the leachability of both zinc and iron could be significantly enhanced.

The different reactivity of EAF waste components is reflected in their leaching kinetics. Zoraga et al. [49] showed that ZnO, ZnFe_2_O_4_, and Fe_2_O_3_ follow distinct kinetic models in nitric acid HNO_3_. ZnO reacts too rapidly to determine kinetics, franklinite dissolution fits a pseudohomogeneous reaction model, and iron oxide is best described by the shrinking core model. Considering these differences, this study applied the shrinking core models to investigate the factors controlling zinc and iron leaching in H_2_SO_4_ and NaOH, assuming homogeneous spherical particles and constant reaction temperature. The following equations [49,50] were examined for the kinetic models of:

(i) A film diffusion-controlled process:

1 − (1 − x)^2/3^ = kt (1)

(ii) A chemical reaction-controlled process:

1 − (1 − x)^1/3^ = kt (2)

(iii) A solid product layer-controlled process:

1 − 3(1 − x)^2/3^ + 2(1 − x) = kt (3)

(iv) A mixed controlled process, i.e., a combination of film diffusion and chemical reaction:

(1 − x) ^−1/3^ + 1/3 ln(1 − x) − 1 = kt (4)

where: x—fractional conversion of zinc or iron, k—reaction rate constant, t—reaction time.

Furthermore, a first-order pseudohomogeneous reaction model was considered, following the dependence below [49,50]:

−ln(1 − x) = kt(5)

**Figure 20:**
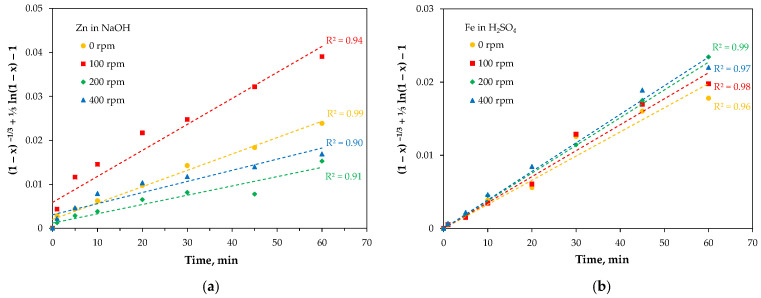
Kinetic model relationship for leaching of: (**a**) Zn in NaOH, (**b**) Fe in H_2_SO_4_.

The dependencies (1)–(5) were plotted in the corresponding coordinate systems. It was found that zinc dissolution in acid occurs so rapidly that it was impossible to accurately determine the reaction model, and no linear fit of the data could be obtained in any case. Similarly, it was not possible to determine the kinetics of iron leaching in NaOH due to the lack of reaction between the components. Consequently, the reaction models for zinc leaching in NaOH and iron leaching in H_2_SO_4_ were established, yielding the best linear fits for a mixed-controlled model (Figure 20). The rate constants were 0.002–0.006 min^−1^ for zinc leaching in NaOH and 0.004 min^−1^ for iron leaching in H_2_SO_4_.

The kinetic evaluation of the reactions during the leaching of EAF waste shows that the reaction types do not align with either chemical or diffusion-based kinetic models of dissolution. Instead, the leaching processes are controlled by interface transfer and diffusion across the solid product layer (like slag component and/or secondary precipitates detected in the solid residues—Figure 16), both of which influence the reaction rate. Compared to the leaching of zinc and iron from pure EAF dust, this behavior is different, but it should be noted that the material was not typical dust and contained slag-like particles. For example, Kukurguya and Havlik [51] concluded that zinc leaching from EAFD using 0.5–1 M H_2_SO_4_ can occur in two steps. The first rate-limiting step was accepted as the diffusion of acid during the reaction with ZnO, while the second step, where acid reacts with ZnFe_2_O_4_, was controlled by the chemical reaction. In contrast, iron dissolved more slowly than zinc, with its rate-limiting step being the chemical reaction. In turn, kinetic studies of direct EAFD leaching in 2.5 M NaOH solution indicated that the reaction was chemically controlled following the shrinking core model [52].

Finally, it is worth noting some aspects related to the milling process. The tungsten carbide balls used in the experiments have a Vickers hardness significantly higher than that of the EAF waste components. After milling, no damage or surface scratches were detected, and no significant mass loss of the balls was observed. For example, at a milling rate of 400 rpm, the total mass change for 10 balls was 0.002 g, corresponding to 0.003% of their total mass, which is essentially within the measurement error. The EDS analysis of the EAF waste, conducted before and after milling at various rates, revealed no tungsten contamination, as no W peaks were detected, confirming the appropriate selection of the milling media.

On the other hand, the introduction of a high-energy milling step affects the economic viability of the entire hydrometallurgical treatment of EAF waste. Although subsequent zinc recovery steps, such as solution purification and final product recovery, were not performed, an additional energy demand for the pretreatment operation can be expected. Assuming a 10 g sample and using the experimentally determined power consumption values, it was calculated that for rotation speeds of 100–400 rpm, the energy consumption ranged from 49 to 63 kWh/kg. It should be emphasized that these data refer to laboratory-scale conditions, and further potential application of high-energy planetary milling for mechanical activation requires additional development, including optimization of milling time, selection of milling additives (e.g., water versus isopropanol), and consideration of chemical additives to induce mechanochemical changes in the refractory franklinite.

## 4. Materials and Methods

Industrial EAF waste was obtained from an undisclosed waste source in Poland. The raw material was characterized in terms of particle size distribution using sieve analysis (five fractions with particle sizes ranging from below 0.125 mm to over 1 mm) and chemical composition. The pre-ground material (for 1 min, laboratory roller-ring mill, Test-Chem, Radlin, Poland) was mechanically activated in a planetary high-energy ball mill (Micro Mill Pulversite 7, Fritsch, Idar-Oberstein, Germany) at different rotation rates (100–400 rpm) and the same conditions of time (4 h; milling sequence: 15 min milling/5 min break), balls (WC balls, 10 mm in diameter, ball-to-EAFD mass ratio 7.5:1) and batch (10 g EAF waste with 2 g water). To determine the power consumption (in watts), measurements were performed using an ORNO OR-WAT-419 plug-in meter (Orno-Logistic Ltd., Gliwice, Poland), which allowed for the calculation of the amount of electrical energy consumed by the device over a given period and providing insights into energy efficiency of the milling. After milling was completed, the resulting powder was carefully collected, dried in glass crystallizer in vacuum drying oven (DZ-1BC II, Chemland, Stargard, Poland), and subsequently used for further investigations.

Both the raw and milled samples were analyzed for morphology as well as chemical and phase composition. Sequential analysis of all samples was performed to determine association of main metals with water-soluble, ion-exchangeable, acid-soluble, reducible, oxidizable, and residual fractions. Sequential extraction of 0.5 g of the EAFD powders was performed according to the scheme shown in Figure 11, following a modified procedure adapted from [42,46]. In each step, 20 cm^3^ of solution was used. The suspensions were subjected to ultrasonic treatment for 1 h at 30 °C, followed by phase separation via centrifugation (5000 rpm, 5 min). Residual solids from each step were water washed and then treated with appropriate leaching reagents in subsequent extraction stages. The final solid residues were water-washed, dried and analyzed by X-ray diffractometry.

The leaching experiments were carefully conducted according to a pre-established methodology, with the reported results representing averaged values. Leaching tests for the raw and milled materials were conducted in 200 cm^3^ of 2 M H_2_SO_4_ or 4 M NaOH at 60 °C, with a liquid-to-solid ratio of 50 and magnetic stirring of 400 rpm. During the 1 h leaching period, 2 cm^3^ aliquots of the solution were taken at regular intervals to determine metal ion concentrations. After leaching, the solid residues were washed, dried, and subjected to phase analysis.

Chemical analysis of the solid phases was carried out using wet digestion in hot aqua regia (2 h, 140 °C) in microwave digestion system (Magnum II, ERTEC, Wrocław, Poland), followed by metal ion determination in obtained solutions using microwave plasma atomic emission spectroscopy (MP-AES 4200, Agilent, Santa Clara, CA, USA). The same method was used for analysis of metal ion concentrations in all solutions. Phase composition of solid samples was analyzed by X-ray diffraction using CuK_α_ radiation (Miniflex 600 diffractometer, Rigaku, Tokyo, Japan). The morphology of the raw and milled EAFD and local metal distribution (point analysis, mapping) was examined using a scanning electron microscope equipped with energy dispersive X-ray spectroscopy (JCM-6000 Plus, JEOL Ltd., Tokyo, Japan).

The reported values represent averages of triplicate experimental data (*n* = 3).

## 5. Conclusions

The mechanical treatment of EAF waste through high-energy ball milling affects the structural properties, metal speciation and leachability. Although, the effect of milling rate on metal extraction is not particularly spectacular, yet it is unquestionable. Increasing the milling intensity results in a reduction in particle size but improves the accessibility of zinc for leaching processes only to a limited extent. At the lowest milling rate (100 rpm, 49 kWh/kg), mechanical activation enhanced zinc dissolution (by about 10%, up to 78% in H_2_SO_4_ and up to 55% in NaOH), likely due to the reduction in particle size, increased surface roughness, and the release of mineral components, making them more accessible for attack by strong acid or base solutions. In contrast, at higher milling rates (200 or 400 rpm, 50 or 63 kWh/kg), secondary synthesis of zinc ferrite occurred, reducing the efficiency of zinc leaching. The leaching kinetics of the main components of the EAF waste seems to be controlled by both interface chemical reaction and diffusion across the solid product layer.

This research study paves the way for optimizing milling parameters to enhance zinc leachability while minimizing the formation of undesirable secondary phases such as franklinite. At the same time, the experimental results raised further research questions, including whether ion substitution of Fe^2+^ by Zn^2+^ in magnetite can occur during high-energy milling to form ZnFe_2_O_4_ (as spinel or inverse spinel), whether a ZnFe_2_O_4_–Fe_3_O_4_ solid solution can be generated, and whether calcium ferrite may react with ZnO to produce franklinite by substitution of Ca^2+^ ion. Another important aspect that has emerged is the development of methodologies for the quantitative determination of individual phases, whether by chemical analysis or diffraction-based approaches (e.g., the Rietveld method), as these remain essential for a deeper understanding of the changes in the complex composition of milled EAF waste.

## Data Availability

The original contributions presented in this study are included in the article. Further inquiries can be directed to the corresponding author(s).

## Abbreviations

The following abbreviations are used in this manuscript:

EAF	Electric arc furnace
EAFD	Electric arc furnace dust
EDS	Energy dispersive X-ray spectroscopy
